# Cellular senescence promotes endothelial activation through epigenetic alteration, and consequently accelerates atherosclerosis

**DOI:** 10.1038/s41598-021-94097-5

**Published:** 2021-07-16

**Authors:** Sakiko Honda, Koji Ikeda, Ryota Urata, Ekura Yamazaki, Noriaki Emoto, Satoaki Matoba

**Affiliations:** 1 Department of Cardiology, Graduate School of Medical Science, Kyoto Prefectural University of Medicine, 465 Kajii, Kawaramachi-Hirokoji, Kamigyo, Kyoto 602-8566 Japan; 2 Department of Epidemiology for Longevity and Regional Health, Graduate School of Medical Science, Kyoto Prefectural University of Medicine, 465 Kajii, Kawaramachi-Hirokoji, Kamigyo, Kyoto 602-8566 Japan; 3 Laboratory of Clinical Pharmaceutical Science, Kobe Pharmaceutical University, 4-19-1 Motoyamakita, Higashinada, Kobe 658-8558 Japan

**Keywords:** Experimental models of disease, Translational research, Cardiovascular biology

## Abstract

Senescent vascular cells are detected in atherosclerotic lesion, and its involvement in the development of atherosclerosis has been revealed; however, whether and the mechanism by which endothelial cell (EC) senescence is causally implicated in atherosclerosis remains unclear. We here investigate a role of EC senescence in atherosclerosis by utilizing EC-specific progeroid mice that overexpress the dominant negative form of telomeric repeat-binding factor 2 under the control of the Tie2 or vascular endothelial cadherin promoter. EC-specific progeria accelerated atherosclerosis in mice with target deletion of ApoE. Mechanistically, senescent ECs were markedly sensitive for inflammation-mediated VCAM-1 induction, leading to enhanced monocyte adhesion. Inhibition of NF-κB signaling abolished the enhanced inflammatory responses in senescent ECs, while NF-κB nuclear translocation in response to TNF-α were similar between young and senescent ECs. We found a higher association of VCAM-1 gene with active histone H3 trimethylated on lysine 4, leading to increased NF-κB accessibility in senescent ECs. Our data revealed that EC cellular senescence causes endothelial hyper-inflammability through epigenetic alteration, which consequently accelerates atherosclerosis. Therefore, EC senescence is a promising therapeutic target for the prevention and/or treatment of atherosclerotic disease in elderly population.

## Introduction

Population aging poses a global health issue, especially in the developed countries. Elder people shows higher morbidity and mortality in various diseases such as stroke, heart attack, heart failure, metabolic disease, and cognitive disease^1^. Recently, crucial roles of cellular senescence have been reported in these age-related diseases^2, 3^. Many stresses including telomere dysfunction, oxidative stress, DNA replication stress, mitochondria dysfunction, and oncogene activation cause cellular senescence^4–6^. A striking feature of cellular senescence is a stable cell cycle arrest, which prohibit the replication of damaged cells and consequently limit the tissue damage and resist tumorigenesis in the short-term^7^. However, senescent cells become harmful in the long term, especially through secreting soluble factors including cytokines, chemokines, and matrix proteases, called senescence-associated secretory phenotype (SASP)^8, 9^. Eliminating senescent cells prevents age-related organ dysfunction in the heart and kidney, and notably, expands the lifespan in mice^10–12^. Also, senescent cell-depletion and senolytic agents that preferentially kill senescent cells improved physical function and showed beneficial effects in age-related diseases such as osteoarthritis and atherosclerosis^13–15^. These findings strongly suggest the critical and causative role of cellular senescence in age-related diseases.

Blood vessels constitute vascular networks throughout the body, and exist in all organs. In addition to provide a route for oxygen and nutrients transportation, blood vessels also contribute to the maintenance of tissue homeostasis, especially through endothelial cell-mediated angiocrine^16–18^. Vascular cells, namely endothelial and smooth muscle cells, become senescent as well as other types of cells with age^19, 20^. We recently generated endothelial progeroid mice in which endothelial cells (ECs) are specifically senescent by overexpressing the dominant negative form of telomere repeat-binding factor 2 (TERF2) in ECs^21^. Interestingly, endothelial progeria impaired metabolic health in mice at young age by inducing adipocyte premature senescence through the SASP.

Senescence of vascular cells is involved in age-related vascular dysfunction including atherosclerosis. Senescent vascular cells have been detected in atherosclerotic plaque, and eliminating senescent cells prevented the progression of atherosclerosis^14, 19^. Also, crucial and causative roles of vascular smooth muscle cell senescence in atherosclerosis have been reported^20^. However, a role of EC senescence in atherogenesis remains to be elusive, though potential role of senescent EC in atherosclerosis was reported^22^. By utilizing the unique endothelial progeroid mice, we identified EC senescence promotes atherosclerosis potentially through EC hyper-inflammability due to epigenetic alteration.

## Results

### EC specific progeria accelerates atherosclerosis

**Figure 1:**
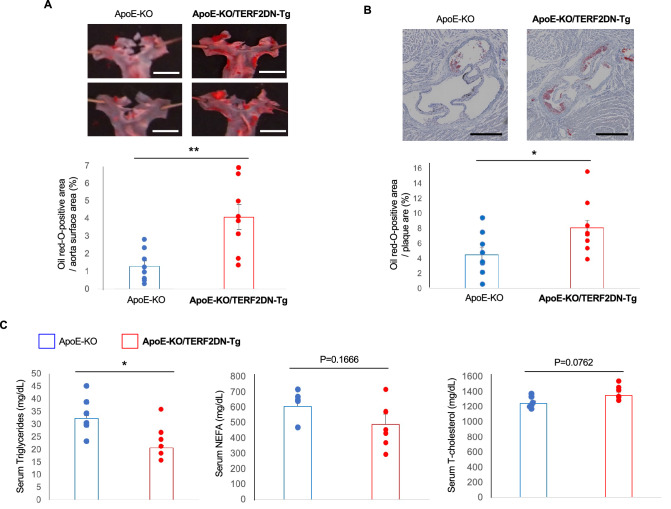
Atherosclerosis was accelerated in EC-specific progeroid mice. (**A**) Typical images for oil red-O staining of enface thoracic aorta isolated from ApoE-KO and ApoE-KO/Tie2-TERF2DN-Tg mice fed high cholesterol diet for 2 weeks are shown. Atherosclerotic plaque was augmented in EC-specific progeroid mice as compared to that in littermate ApoE-KO mice (n = 8 each). Bars: 2 mm. (**B**) Representative images of oil red-O staining in aortic sinus prepared from ApoE-KO and ApoE-KO/Tie2-TERF2DN-Tg mice fed high cholesterol diet for 2 weeks. Atherosclerotic progression was accelerated in EC-specific progeroid mice (n = 8 each). Bars: 500 μm. (**C**) Serum lipid profiles in ApoE-KO and ApoE-KO/Tie2-TERF2DN-Tg mice fed high cholesterol diet for 2 weeks (n = 6 each). Data are presented as mean ± SEM. *p < 0.05 and **p < 0.01.

Aging is one of the most important risk factors for atherosclerotic disease; however, it remains unclear whether and by which mechanism EC senescence is causally involved in the progression of atherosclerosis. We recently generated mice in which ECs are specifically senescent due to DNA damage (Tie2-TERF2DN-Tg mice)^21^. The transgene expression in ECs but not in non-ECs isolated from the lungs of Tie2-TERF2DN-Tg mice was confirmed (Supplementary Fig. S1). We newly generated athero-prone ApoE-deficient mice in which ECs are specifically senescent (ApoE-KO/Tie2-TERF2DN-Tg) to analyze a role of EC senescence in the progression of atherosclerosis. Because EC injury is an initial event for atherosclerosis, we assessed atherosclerosis at an early stage by feeding mice with high cholesterol diet (HCD) for 2 weeks. Only small atherosclerotic plaques were detected in the aortic arch of ApoE-KO mice after 2-week of HCD (Fig. 1A). Of note, atherosclerotic plaque formation was significantly enhanced in ApoE-KO/Tie2-TERF2DN-Tg mice comparing to that in ApoE-KO mice fed a HCD for 2 weeks (Fig. 1A,B). Serum cholesterol and fatty acid levels were not different between these mice, while serum triglycerides were reduced in ApoE-KO/Tie2-TERF2DN-Tg mice (Fig. 1C). These data indicate a crucial and causative role of EC senescence in the progression of atherosclerosis in vivo.

### Generation of another EC progeroid mice

**Figure 2:**
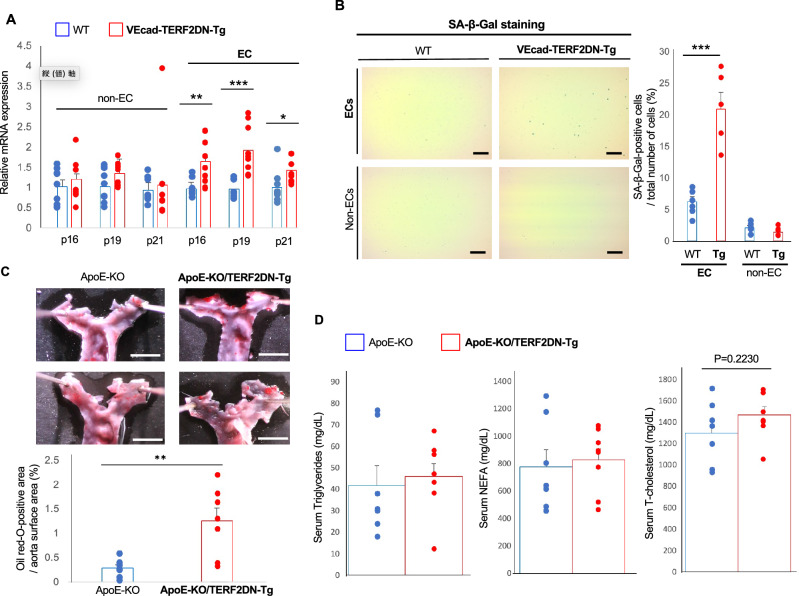
Validation of accelerated atherosclerosis using a new line of EC-specific progeroid mouse. (**A**) CDK inhibitors expression in EC and non-EC isolated from the lungs of WT and VEcad-TERF2DN-Tg mice (n = 9 each). (**B**) SA-β-Gal staining in ECs and non-ECs isolated from the lungs of WT or VEcad-TERF2DN-Tg mice. The number of SA-β-Gal-positive cells was counted (n = 6). Bars: 100 μm. (**C**) Typical images for oil red-O staining of enface thoracic aorta isolated from ApoE-KO and ApoE-KO/VEcad-TERF2DN-Tg mice fed high cholesterol diet for 2 weeks are shown. Atherosclerotic plaque formation was enhanced in EC-specific progeroid mice (n = 7 each). Bars: 2 mm. (**D**) Serum lipid profiles in ApoE-KO and ApoE-KO/VEcad-TERF2DN-Tg mice fed high cholesterol diet for 2 weeks (n = 7 for ApoE-KO; n = 8 for ApoE-KO/TERF2DN-Tg). Data are presented as mean ± SEM. *p < 0.05, **p < 0.01, and ***p < 0.001.

We used Tie2 promoter to generate EC-specific progeroid mice. However, Tie2 promoter is known to be active in hematopoietic cells including macrophages that play critical roles in the progression of atherosclerosis. To exclude a possible effect of hematopoietic cell senescence, we newly generated another EC-specific progeroid mice using vascular endothelial cadherin promoter that is largely inactive in hematopoietic cells postnatally^23^ (VEcad-TERF2DN-Tg). VEcad-TERF2DN-Tg mice were viable and fertile as well as Tie2-TERF2DN-Tg mice. We then confirmed EC-specific senescence in this new line of EC-specific progeroid mouse. We detected significant increase in cyclin-dependent kinases expression and enhanced senescence-associated β-galactosidase activity in ECs, while none of them was detected in non-ECs (Fig. 2A,B). Also, the transgene expression in ECs but not in non-ECs isolated from the lungs of VEcad-TERF2DN-Tg mice was confirmed (Supplementary Fig. S1). Furthermore, immunohistochemistry for p16 showed stronger staining in aortic ECs in VEcad-TERF2DN-Tg than in aortic ECs in WT mice, suggesting the accelerated cellular senescence in ECs of the Tg mice (Supplementary Fig. S2). Subsequently, we generated EC-specific progeroid ApoE-KO mice using the VEcad-TERF2DN-Tg mice, and fed them with HCD for 2 weeks. Similar to ApoE-KO/Tie2-TERF2DN-Tg mice, ApoE-KO/VEcad-TERF2DN-Tg mice showed exacerbated atherosclerosis as compared to ApoE-KO mice after 2-week of HCD feeding (Fig. 2C). Serum lipid profiles were similar between these mice (Fig. 2D).

### Cellular senescence causes EC hyper-activation in response to inflammation

**Figure 3:**
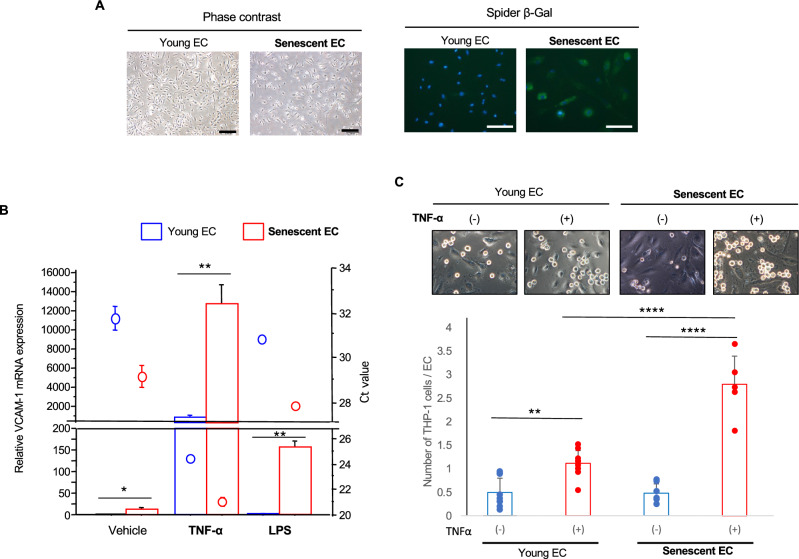
Senescent EC is hypersensitive to inflammatory stimuli. (**A**) Replicative senescent HUVECs were prepared. Senescent ECs showed flattened and enlarged morphology, and senescence-associated β-Gal activity. Bars: 200 μm. (**B**) Quantitative PCR analysis for VCAM-1 in ECs (n = 7 each for vehicle group; n = 3 each for TNF-α and LPS groups). Young control and senescent ECs were treated with 1 ng/ml TNF-α or 5 ng/ml LPS for 24 h. (**C**) Young control and senescent ECs were treated with 1 ng/ml TNF-α for 24 h, and then THP-1 monocyte adhesion was examined (n = 9 for young ECs: n = 5–6 for senescent ECs). Bars: 100 μm. Data are presented as mean ± SEM. *p < 0.05, **p < 0.01, and ****p < 0.0001.

To explore the mechanisms by which senescent ECs accelerate atherosclerosis, we prepared replicative senescent ECs in vitro (Fig. 3A). Because an initial event in atherosclerosis is monocyte adhesion onto ECs, which is mediated largely through endothelial cell adhesion molecules (CAM)^24^, especially vascular cell adhesion molecule (VCAM)-1^25, 26^, we examined VCAM-1 expression in young and senescent ECs. Basal VCAM-1 expression levels were minimal assessed by the Ct value, but the relative expression levels were significantly higher in senescent ECs (Fig. 3B). Inflammation is the major trigger for activation and CAMs induction in ECs; therefore, we treated cells with relatively weak inflammatory stimuli using TNF-α and LPS in low concentration. These weak inflammatory stimuli were sufficient to mediate a significant induction of VCAM-1 expression in young ECs (*p* = 0.0004 for TNFα-treated cells; *p* = 0.0121 for LPS-treated cells). Of note, robust induction of VCAM-1 was observed in senescent ECs (p < 0.0001 for both TNFα- and LPS-treated cells), and the expression levels of VCAM-1 were markedly high in senescent ECs as compared to those in young ECs after inflammatory stimuli (Fig. 3B).

We then examined monocyte adhesion onto ECs using THP-1 human monocyte cells. At basal condition, monocyte adhesion onto senescent ECs did not differ from that onto young ECs (Fig. 3C). Although relative expression levels of VCAM-1 were higher in senescent ECs than in young ECs at basal condition, the absolute amount of VCAM-1 might not be sufficient to induce monocyte adhesion, at least in our experimental condition. When treated with TNF-α, monocyte adhesion was enhanced in both young and senescent ECs (Fig. 3C). Consistent with the robust VCAM-1 induction in response to inflammatory stimuli, monocyte adhesion was significantly enhanced in senescent ECs comparing to that in young ECs after treatment with TNF-α (Fig. 3C). These data strongly suggest that senescent ECs are highly inflammable, and consequently mediate enhanced monocyte adhesion onto the EC layer.

### NF-κB pathway is involved in the hyper-inflammability in senescent ECs

**Figure 4:**
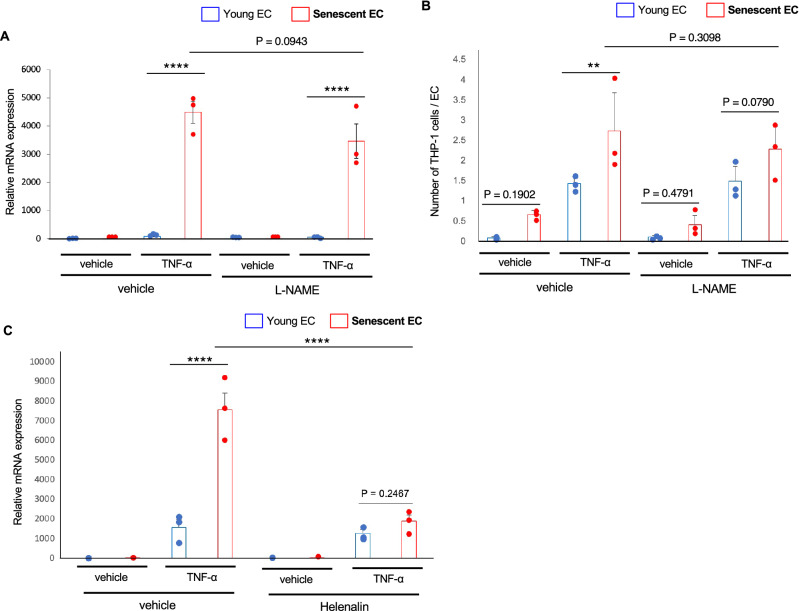
NF-κB signaling is critically involved in the hyper-inflammability in senescent ECs. (**A**) Inhibition of NOS using L-NAME did not abolish the enhanced VCAM-1 induction in response to TNF-α in senescent ECs (n = 3 each). (**B**) Inhibition of NOS using L-NAME did not abrogate the enhanced monocyte adhesion in senescent ECs treated with TNF-α (n = 3 each). (**C**) Inhibition of NF-κB signaling using helenalin abolished the enhanced VCAM-1 induction in response to TNF-α in senescent ECs (n = 3 each). Data are presented as mean ± SEM. **p < 0.01 and ****p < 0.0001.

EC-derived nitric oxide (NO) is crucially involved in the pathogenesis of atherosclerosis^27^, and endothelial NO synthase (eNOS) expression has been reported to decrease during aging in ECs^28^. Therefore, we investigated a possible role of eNOS in the hyper-inflammability in senescent ECs. NOS inhibition by L-NAME did not significantly affect the TNF-α-mediated VCMA-1 induction in ECs, and the robust VCAM-1 induction in senescent ECs remained after the treatment with L-NAME (Fig. 4A). Consistently, L-NAME treatment did not show significant impact in monocyte adhesion onto ECs, while monocyte adhesion showed only tendency toward increase in senescent ECs in the presence of L-NAME (Fig. 4B). These data collectively suggest a minor role of NO pathway in the hyper-inflammability in senescent ECs.

### NF-κB signaling is enhanced in senescent ECs through epigenetic alteration

**Figure 5:**
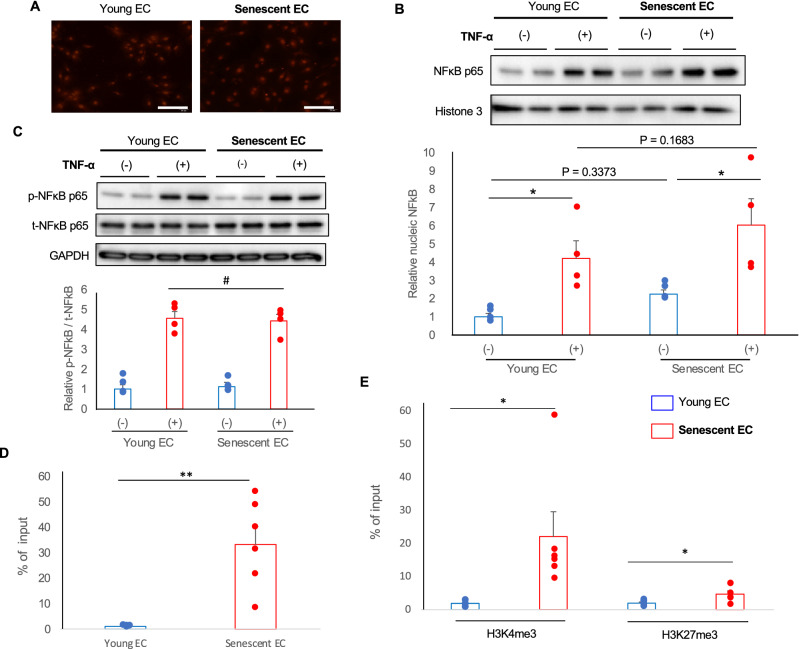
Epigenetic alteration causes the enhanced NF-κB signaling in senescent ECs. (**A**) Nuclear translocation of NF-κB p65 in response to TNF-α was assessed by immunocytochemistry. Bars: 200 μm. (**B**) Nuclear accumulation of NF-κB p65 in response to TNF-α was assessed by immunoblotting using nucleus extracts (n = 4 each). Blots cropped from different parts of the same gel were used. Uncropped blots images are shown in the Supplementary Fig. S3A. (**C**) Activation of NF-κB in response to TNF-α was assessed by immunoblotting for phospho-NFκB p65 (n = 4 each). For p-NF-κB and GAPDH, blots cropped from different parts of the same gel were used. For t-NF-κB, a blot prepared from the different gel (that was prepared using the same protein samples at the same time in parallel) was used. Uncropped blots images are shown in the Supplementary Fig. S3B. (**D**) NF-κB p65 binding to the promoter region for VCAM-1 was assessed by ChIP assay in young and senescent ECs treated with TNF-α (n = 6 each). (**E**) VCAM-1 gene association with histone H3 with activating (K4) or repressing (K27) trimethylation (me3) was assessed by ChIP assay (n = 6 each). Data are presented as mean ± SEM. *p < 0.05, and **p < 0.01. #, not significant.

Because NF-κB governs the pro-inflammatory response in EC^29^, we then examined a role of NF-κB pathway in the hyper-inflammability in senescent ECs. Inhibition of NF-κB signaling by helenalin dramatically reduced VCAM-1 expression in senescent ECs treated with TNF-α to the levels similar to that in young ECs (Fig. 4C). These data indicate that enhanced NF-κB signaling is causally involved in the hyper-inflammability in senescent ECs. To further analyze the underlying mechanism, we assessed the NF-κB nuclear translocation in response to TNF-α using immunocytochemistry for NF-κB p65. NF-κB p65 nuclear translocation were readily detectable even in young ECs treated with TNF-α in low concentration, and there was no significant difference of the NF-κB p65 nuclear translocation between young and senescent ECs after treatment with TNF-α (Fig. 5A). Immunoblotting for NF-κB p65 in nuclear extracts also showed similar nuclear accumulation of NF-κB p65 in young and senescent ECs treated with TNF-α (Fig. 5B). Furthermore, activation of NF-κB p65 in response to TNF-α also showed no difference between young and senescent ECs (Fig. 5C).

We then assessed the accessibility of NF-κB p65 to the VCAM-1 promoter by using chromatin immunoprecipitaion assay. Accordingly, we found that NF-κB p65 binding to the promoter region for VCAM-1 was substantially enhanced in senescent ECs as compared to that in young ECs (Fig. 5D). These data suggest that epigenetic alterations during aging is causally involved in the hyper-inflammability in senescent ECs.

Open/close chromatin status is critical for transcription factor accessibility towards target genes, and histone methylation is one of the major regulators for chromatin rigidity^30^. Trimethylation of histone H3 on the lysine at position 4 (H3K4me3) is associated with an open chromatin structure, whereas trimethylation of histone H3 on the lysine 27 (H3K27me3) is associated with a closed chromatin^30^. We therefore explored the association of VCAM-1 gene with H3K4me3 and H3K27me3 in young and senescent ECs. VCAM-1 gene association with activating H3K4me3 was substantially enhanced, while less increased association with repressing H3K27me3 was detected in senescent ECs comparing to those in young ECs (Fig. 5E). These data strongly suggest that VCAM-1 gene is highly associated with nucleosome in which histone H3 are trimethylated on lysine 4, namely open chromatin, in senescent ECs, leading to the higher accessibility and translational activity for NF-κB in senescent ECs.

### Atherosclerotic plaque property is affected by EC senescence

**Figure 6:**
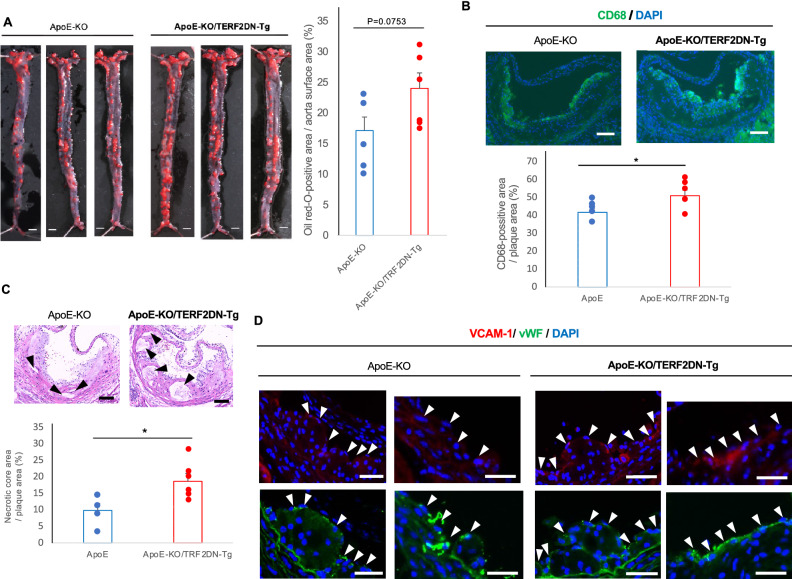
EC senescence affects the plaque characteristics. (**A**) En face analysis of the aorta in ApoE-KO and ApoE-KO/VEcad-TERF2DN-Tg mice fed a HCD for 8 weeks. Atherosclerotic plaques were quantitatively analyzed (n = 5 for ApoE-KO; n = 6 for ApoE-KO/TERF2DN-Tg). Bars: 1 mm. (**B**) Immunohistochemistry for CD68 in aortic sinus sections of ApoE-KO and ApoE-KO/VEcad-TERF2DN-Tg mice fed a HCD for 8 weeks. CD68-positive areas were quantified (n = 5 for ApoE-KO; n = 6 for ApoE-KO/TERF2DN-Tg). Bars: 100 μm. (**C**) H-E staining of aortic sinus sections of ApoE-KO and ApoE-KO/VEcad-TERF2DN-Tg mice fed a HCD for 8 weeks. Arrowheads indicate the necrotic core. The size of necrotic core was measured (n = 5 for ApoE-KO; n = 6 for ApoE-KO/TERF2DN-Tg). Bars: 100 μm. (**D**) Immunohistochemistry for VCAM-1 in aortic sinus sections of ApoE-KO and ApoE-KO/VEcad-TERF2DN-Tg mice fed a HCD for 2 weeks. Immunohistochemistry for vWF was performed using the serial sections to identify ECs. Arrowheads indicate the vWF-positive endothelium on the plaque surface. Bars: 100 μm. Data are presented as mean ± SEM. *p < 0.05.

To further investigate a role of senescent ECs in the pathogenesis of atherosclerosis, we examined the plaque properties through histological analysis in aortic sinus sections. We treated the mice with HCD for 8 weeks, and then assessed the atherosclerosis. Atherosclerotic plaques, assessed by the en face analysis showed tendency toward increase in ApoE-KO/VEcad-TERF2DN-Tg mice comparing to that in ApoE-KO mice fed a HCD for 8 weeks (Fig. 6A). Of note, macrophage infiltration in the plaques, assessed by immunohistochemistry for CD68 was augmented in ApoE-KO/VEcad-TERF2DN-Tg mice as compared to that in ApoE-KO mice fed a HCD for 8 weeks (Fig. 6B). Furthermore, augmented necrotic core formation in the plaques was detected in ApoE-KO/VEcad-TERF2DN-Tg mice comparing to that in ApoE-KO mice after 8 weeks of HCD (Fig. 6C). These results collectively suggest that EC senescence accelerates the atherosclerosis from an early stage, and subsequently affect the plaque characteristics at an advanced stage.

To explore a role of hyper-inflammability in senescent ECs in the progression of atherosclerosis, we analyzed the VCAM-1 expression in the endothelium of the plaque surface at an early stage. Immunohistochemistry for VCAM-1 showed enhanced fluorescence signals in the plaque surface ECs of ApoE-KO/VEcad-TERF2DN-Tg mice comparing to that in ApoE-KO mice after 2 weeks of HCD (Fig. 6D). These data support a detrimental role of hyper-inflammability in senescent EC in the progression of atherosclerosis by promoting the macrophage recruitment into the plaques at least partly through enhanced VCAM-1 expression.

## Discussion

Atherosclerosis is a primary cause for cardiovascular disease including stroke and heart attack. Aging is closely associated with the morbidity and mortality of atherosclerotic disease, and vascular aging has been considered to play a role in the progression of atherosclerosis. ECs constitute a single inner layer of blood vessels, and regulate vascular functions such as vasodilation and angiogenesis. It has been reported that cellular senescence in ECs causes reduced vasodilation capacity and impaired neovessel formation, at least partially through reduction in eNOS and Bcl-2 expression during aging^28, 31^. However, little is known about a role of EC senescence in the pathogenesis of atherosclerosis. In the current study, we identified a positive correlation of EC senescence with the development of atherosclerosis by utilizing the unique EC-specific progeroid mice. We have validated the EC senescence in these EC-specific progeroid mice using ECs isolated from the lungs and by immunohistochemistry for p16 in aorta. More detailed analysis for senescence features in ECs is required to establish these mice as a suitable model for EC senescence studies.

Atherosclerosis is a chronic inflammatory disease of blood vessels that is characterized by atheromatous plaque formation. Various stresses including oxidative stress, low arterial shear stress, and hyperlipidemia cause chronic inflammation and activation in ECs^32, 33^. One of the striking features in activated ECs is high expression of CAMs such as VCAM-1^34^. The CAMs mediate leukocyte-EC interaction that is manifested as rolling and subsequent transendothelial migration of leukocytes, leading to plaque formation^24, 34^. Therefore, hyper-activation in EC accelerates atherosclerosis by enhancing monocyte recruitment into the plaques. In this study, we demonstrated the enhanced adhesion capacity for monocytes in senescent ECs in association with the higher VCAM-1 expression. Because of the poor proliferation in senescent ECs, we had difficulties to prepare the confluent EC monolayer as well as to prepare young and senescent ECs in a similar confluency. Therefore, the number of attached monocytes was normalized with the number of ECs when compare the data between young and senescent ECs. This normalization is not well-standardized, and a caution is needed to interpret the adhesion assay data.

Previous report showed that eNOS plays minimal role in EC activation and CAM expression in ECs^35^. Consistently, NOS inhibition failed to inhibit the enhanced VCAM-1 induction in senescent EC in this study. However, it has been reported that eNOS plays a protective role against atherosclerosis, and that its expression is reduced in senescent ECs^27, 28^. Therefore, eNOS deficiency might be also involved in the causative link between EC senescence and atherosclerosis through the mechanism different from the senescence-associated hyper-activation in EC.

Alteration in DNA methylation, histone post-translational modification and chromatin organization is closely associated with healthspan and lifespan^36^. These epigenetic alterations have been widely accepted as hallmarks of aging, and the aging epigenome provides a link between cellular senescence and senescence-associated cellular dysfunctions^37^. An imbalance of activating and repressive histone modifications leads to dysregulated gene expressions by modulating the access of transcriptional factors to DNA. It has been reported that histone methylations, especially H3K4me3 (activating) and H3K27me3 (repressing) are directly linked to lifespan regulation in many organisms^36^. We found that senescence-associated EC hyper-inflammability is largely due to enhanced NF-κB signaling, whereas NF-κB p65 activation and nuclear translocation in response to inflammatory stimuli were not different between young and senescent ECs. These data urged us to investigate a role of epigenetic alterations in the enhanced NF-κB signaling in senescent ECs. We detected a robust increase in NF-κB p65 binding to VCAM-1 promoter in association with the higher correlation of VCAM-1 promoter region with activating H3K4me3 in senescent ECs comparing to those in young ECs. These data strongly suggest a crucial role of aging epigenome in the hyper-inflammability in senescent ECs. Association of VCAM-1 promoter region with repressing H3K27me3 was also increased in senescent ECs, but the degree of increase was considerably high for H3K4me3, suggesting a relatively open chromatin status for VCAM-1 gene in senescent ECs. In combination with the in vivo and in vitro results, our data provide strong evidence for a causative role of EC senescence in the progression of atherosclerosis through hyper-inflammability due to epigenetic alterations; thus, senescent EC, especially their aging epigenomes is an attractive pharmaco-therapeutic target for the prevention and treatment of atherosclerotic disease.

## Materials and methods

### Materials

Antibodies used were: for NF-κB p65 (Active Motif #39369); phospho-NF-κB p65 (S536) (Cell Signaling Technology #3033); H3K4me3 (TaKaRa #MA304A); H3K27me3 (TaKaRa #MA323A); GAPDH (Sigma #MAB374); Histone-3 (Cell Signaling Technology #4499S); CD68-Alexa488 (Abcam #ab201844); VCAM-1 (Cell Signaling Technology #32653); vWF (Abcam #ab9378); p16 (Abcam #ab192053).

Recombinant human TNF-α (#210-TA) was obtained from R&D systems. L-NAME (#80210) was obtained from Cayman Chemical. LPS (#L2630) was obtained from Sigma. Helenalin (#ab146197) was obtained from Abcam.

### Cell culture

Human umbilical vein endothelial cells (HUVECs) were obtained from LIFELINE Cell Technology. Replicative senescent EC was prepared as previously described^21, 31^. Briefly, HUVECs were passaged at 1:4 ratio for 15–17 times (P18-20) until no obvious growth was observed. P3-5 HUVECs were used as control young cells. Cells were treated with 5 ng/ml LPS or 1 ng/ml TNF-α for 24 h to induce inflammatory responses. In some experiments, cells were treated with TNF-α in the presence of 100 μM L-NAME or 1 μM Helenalin.

### Monocyte adhesion assay

Young and senescent HUVECs were plated on 48-well plate. When reached nearly confluent, 1 × 10^5^ THP-1 cells were added onto the EC layer and incubated for 30 min, followed by gentle washing with PBS twice. Subsequently, cells were observed under phase-contrast microscopy, and remaining THP-1 cells on EC layer were counted. In some experiments, cells were treated with 100 μM L-NAME.

### Quantitative RT-PCR

Quantification of mRNA expression of target genes was performed as previously reported^21^. Briefly, total RNA was extracted from cells or tissues using Trizol (Invitrogen), followed by purification with Direct-zol RNA mini-prep Kit (Zymo Research). Complementary DNA was synthesized from 1 μg of total RNA using the PrimeScript RT Master Mix (TaKaRa). PCR reactions were prepared using KAPA SYBR Fast (KAPA Biosystems), followed by quantitative PCR on Thermal Cycler Dice (TaKaRa). Quantification of gene expression was performed using the delta-delta Ct method, and the target gene expression levels were normalized with β-actin expression levels. The nucleotide sequence of each primer is shown in Table 1.

**Table 1:** The nucleotide sequence of primers.

**Nucleotide sequence of primers for RT-qPCR**	
Human VCAM-1	5′-TTTGACAGGCTGGAGATAGACT-3′
	5′-TCAATGTGTAATTTAGCTCGGCA-3′
TERF2DN-transgene	5′-AAGCTTGGTACCGAGCTCGGATCAGC-3′
	5′-CTCGGATCCTCTCTTTCTTAACAAATCT-3′
GAPDH	5′-CCTTCATTGACCTCAACTACATGG-3′
	5′-CCTGCTTCACCACCTTCTTGAT-3′
**Nucleotide sequence of primers for ChIP**	
VCAM-1 gene promoter region	5′-TGGAACTTGGCTGGGTGTCTGTTAA-3′
	5′-TGCTTTATAAAGGGTCTTGTTGCAGAG-3′

### ChIP assay

ChIP assay was performed using ChIP-IT Express Enzymatic Kit (Active Motif, #53009) as the manufacturers recommended. Cells were treated with 1 ng/ml TNF-α for 11 h before fixation with 1% formaldehyde. After scraping, nuclei were extracted by using Dounce homogenizer, followed by enzyme DNA shearing. Sheared chromatin (~ 20 μg) was immunoprecipitated with either anti-NFκB p65, H3K4me3 or H3K27me3 antibody, and subsequently subjected to the reverse cross-linking. Some sheared chromatin was preserved as an input control. Immunoprecipitated chromatin was subjected to PCR amplification for VCAM-1 promoter gene flanking NF-κB cis elements. Nucleotide sequence of the primer is shown in Table 1.

### Animal study

All experimental protocols were approved by the Ethics Review Committee for Animal Experimentation of Kyoto Prefectural University of Medicine. All researchers have complied with all relevant ethical regulations for animal testing and research, and animal experiments were performed in compliance with ARRIVE (Animal Research: Reporting of In Vivo Experiments) guidelines. Transgenic mice that overexpressed TERF2DN in EC (Tie2-TERF2DN-Tg and VEcad-TERF2DN-Tg) were generated (C57/BL6J background). The plasmid containing the TERF2-∆B-∆M was obtained from Addgene (plasmid #2431)^6^. The plasmid containing the Tie2-promoter and enhancer was a gift from Dr. Thomas N. Sato. The plasmid containing the VEcad-promoter was a gift from Dr. Mochizuki and Dr. Nakaoka (National Cardiovascular Research Center). The Tg mice were propagated as heterozygous Tg animals by breeding with WT C57/BL6J mice.

Mice were housed in designated cages of sufficient size (1–3 mice in one cage) in animal facility in which the temperature and humidity are regulated at ~ 23 °C and ~ 60%, respectively. Mice were maintained under a 12-h light/12-h dark cycle, and fed chow with ad libitum access to water and food. Male mice were used for all the experiments. Mice were fed with a high-cholesterol diet containing 16.5% fat and 1.25% cholesterol (Oriental Yeast, Japan), starting at the age of 10 weeks old, for 2 or 8 weeks. Littermate ApoE-KO mice were always used as controls for ApoE-KO/TERF2DN-Tg mice. For en face analysis, the entire aorta from the heart, extending 5 mm after bifurcation of the iliac arteries and including the subclavian right and left common carotid arteries, was removed, dissected, and stained with oil red-O^38^. The oil red-O-positive atherosclerotic lesion area was measured using the Image J software. For the analysis of the atherosclerotic lesion at the aortic sinus, serial cryosections were prepared from the region of the proximal aorta through the aortic sinuses.

### Isolation of EC from the mouse lungs

ECs were isolated from the lungs of mice as previously described^21^. Briefly, dissociation and homogenization of the lungs were performed using MACS dissociation kit (Miltenyi Biotec, #130-095-927), gentleMACS C tube (#130-093-237), and gentleMACS Dissociators (#130-093-235) according the manufacturer’s protocol. Subsequently, cells were incubated with FcR Blocking reagent for mouse (#130-092-575) for 10 min at 4 °C, followed by incubation with CD146 (LSEC) MicroBeads (#130-092-007) for 15 min at 4 °C. After washing, cells were applied to LS column (#130-042-401) in the magnetic field of MACS separator (#130-042-301). The flow-through medium containing EC-depleted cells were collected, and these cells were used as non-ECs.

### SA-β-Gal and SPiDER-β-Gal staining

SA-β-Gal and SPiDER-β-Gal staining was performed as previously reported^21^. For SA-β-Gal staining, cells were fixed with 4% PFA, and then incubated with SA-β-Gal staining solution (40 mM sodium phosphate, ph 6.0 + 5 mM potassium ferrocyanide + 5 mM potassium ferricyanide + 150 mM NaCl + 2 mM MgCl_2_ + 1 mg/ml X-Gal) at 37 °C. Cells were observed every 1 h during the first 6 h, and subsequently every 4–8 h. When the cells are stained as blue-green visualized under an inverted bright-field microscope, the reaction was terminated by washing with pure water.

For SPiDER-β-Gal staining, cells were fixed with 4% PFA, and then incubated with ~ 0.3 ng/μl SPiDER-βGal (DOJINDO) in Mcllvaine buffer (pH 6.0) for 30 min at 37 °C. Subsequently, cells were observed under fluorescence microscopy.

### Immunohistochemistry

Frozen sections of aortic sinus were incubated with 10% normal donkey serum at room temperature for 1 h for blocking. Subsequently, sections were incubated with first antibody (1:200 for VCAM-1, 1:200 for CD68-Alexa488, and 1:100 for vWF) at 4 °C for overnight, followed by washing in PBS. For VCAM-1 and vWF immunostaining, sections were then incubated with secondary antibody labeled with Alexa-594 and Alexa-488, respectively, at room temperature for 1 h. Finally, sections were mounted with antifade reagent containing DAPI (VECTASHIELD), and analyzed under fluorescence microcopy (KEYENCE BZ-X810).

Frozen section of aorta were incubated in 3% H_2_O_2_ for 10 min, followed by incubation with 5% normal donkey serum for 1 h at room temperature. Subsequently, sections were incubated with avidin- and biotin-blocking buffer (Vector Laboratories) for 15 min each, followed by incubation with anti-p16 antibody (1:100) at 4 °C for overnight. After washing with PBS, sections were incubated with biotinylated secondary antibody (Vector Laboratories, 1:300) for 1 h at room temperature, followed by incubation with VECTASTAIN Elite ABC-HRP reagent (Vector Laboratories) for 30 min. Finally, sections were incubated with ImmPACT DAB Substrate (Vector Laboratories), followed by hematoxylin nuclear staining.

### Statistical analysis

Differences between 2 groups were analyzed by Student’s t test, while differences between 3 or more groups were analyzed by one-way ANOVA with post hoc multiple comparison by Bonferroni/Dunn’s test. p < 0.05 was considered statistically significant. Data are presented as mean ± SE as indicated.

## Supplementary Information

Supplementary Information.

## Data Availability

The authors declare that all data supporting the findings of this study are available within the paper.
