# Supplementary material for: Cellular senescence promotes endothelial activation through epigenetic alteration, and consequently accelerates atherosclerosis
Source: Sci Rep. 2021 Jul 16;11:14608. doi: 10.1038/s41598-021-94097-5 (PMC8285500; doi:10.1038/s41598-021-94097-5)
Supplement: Supplementary file 1 — Supplementary Information. [file 41598_2021_94097_MOESM1_ESM.pdf]

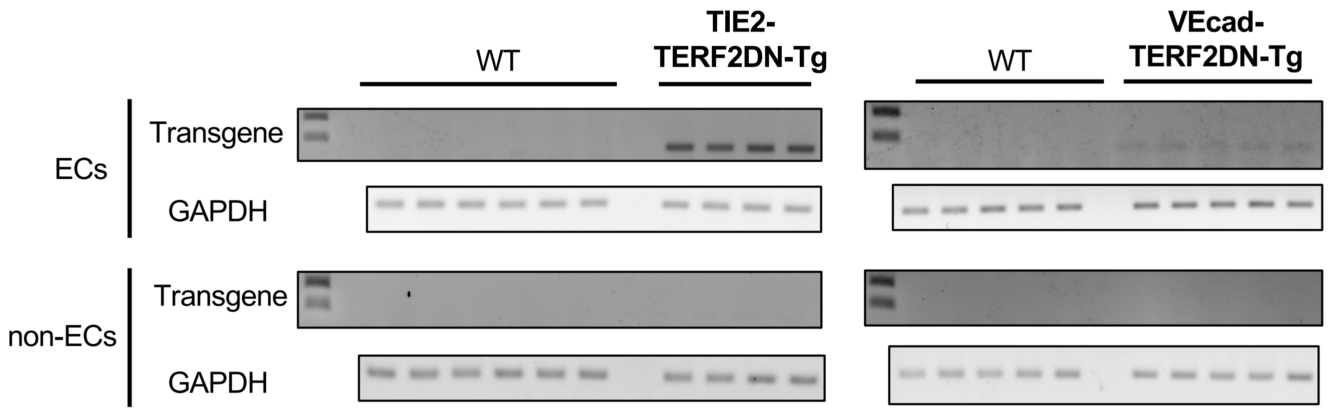

Supplementary Figure-1. Transgene expression in ECs of EC-specific progeroid mouse.
cDNAs were prepared from ECs and non-ECs isolated from the lungs of either WT, TIE2-TERF2DN-Tg, or VEcad-TERF2DN-Tg mice. PCR was performed for these cDNAs using the transgene-specific primers or GAPDH primers. PCR products were run on agarose gel containing ethidium bromide, and the amplified target genes were visualized under ultraviolet irradiation. Uncropped images are shown in Supplementary Fig. 4.

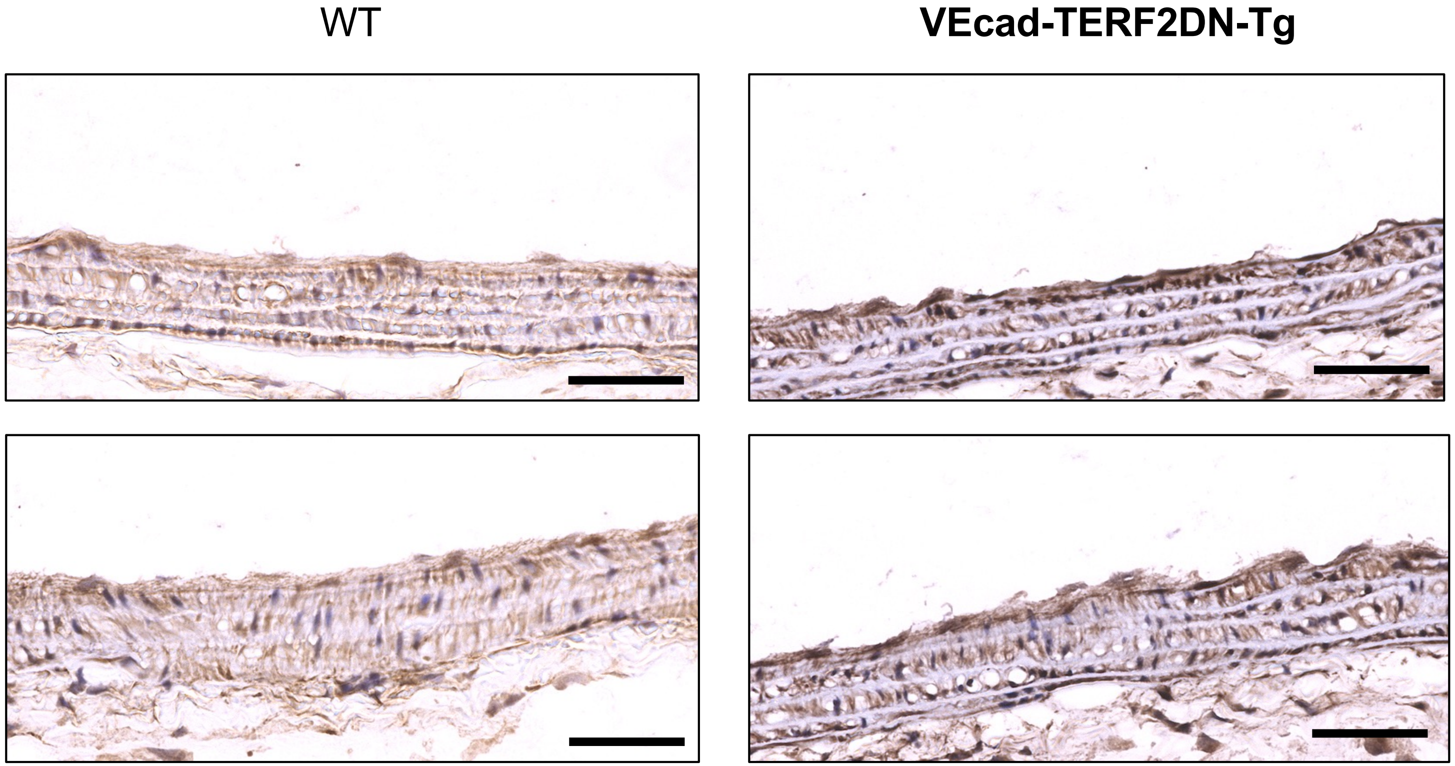

Supplementary Figure-2. Immunohistochemistry for p16 in aorta.
Representative images of immunohistochemistry for p16 in mouse aorta are shown. Aorta was isolated from WT and VEcad-TERF2DN-Tg mice. Bars: 50 μm.

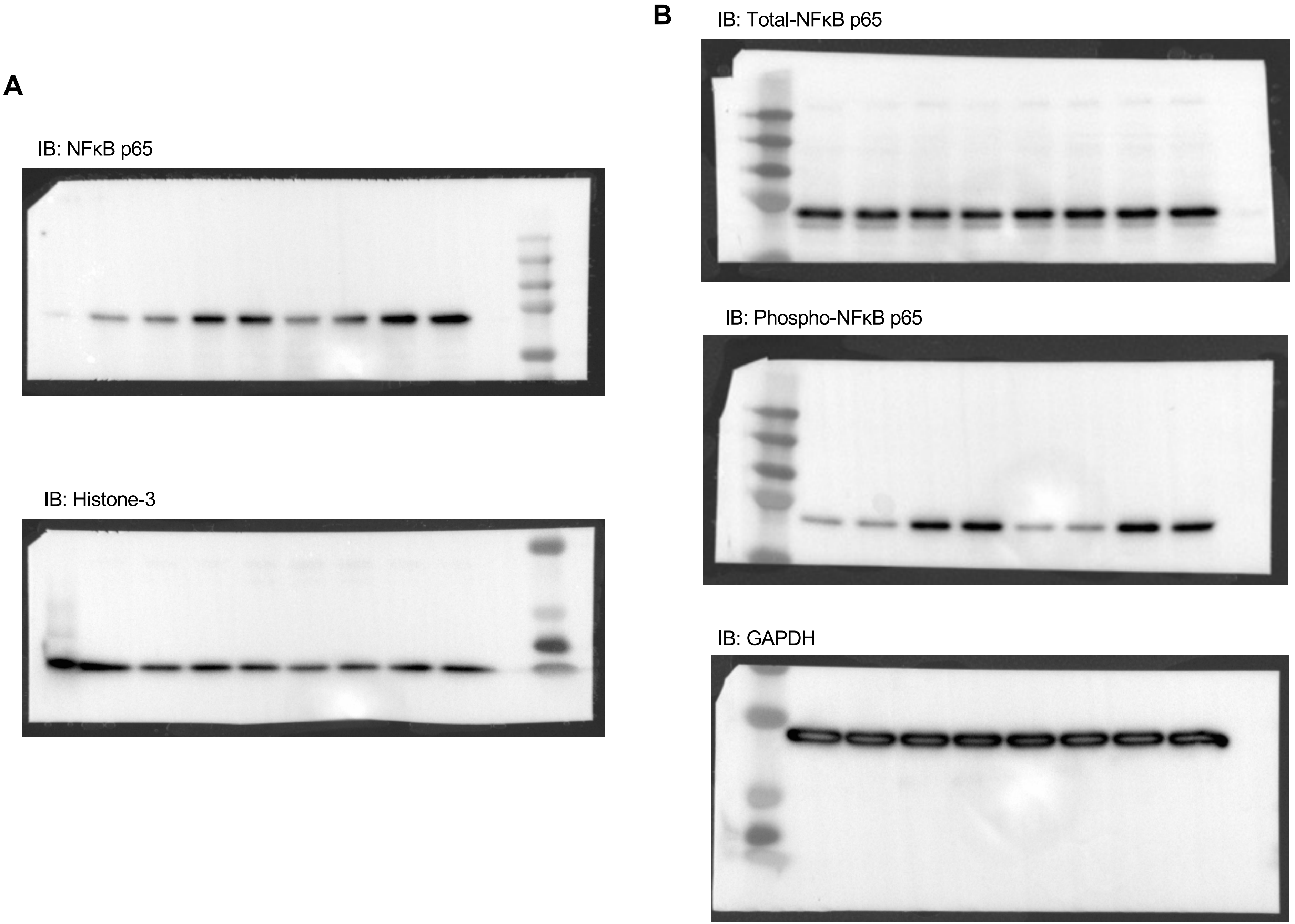

Supplementary Figure-3. Uncropped images for Figure-5.
(**A**) Uncropped images for Figure-5B. (**B**) Uncropped images for Figure-5C.

A

PCR TRANSGENE
ECs TIE2-TRF2DN-Tg, 25 cycles

1 kb ladder marker

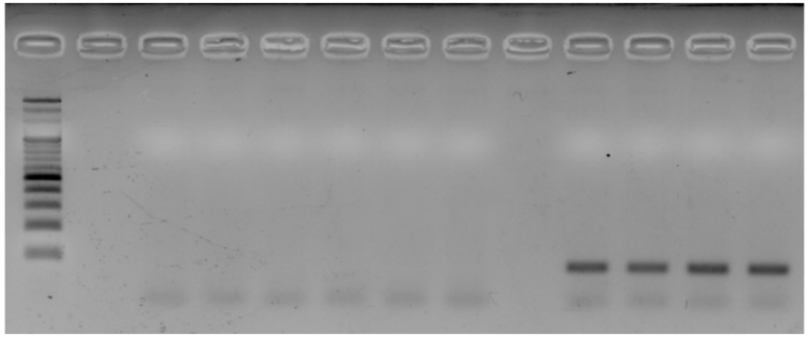

PCR TRANSGENE
Non – ECs TIE2-TRF2DN-Tg, 25 cycles

1 kb ladder marker

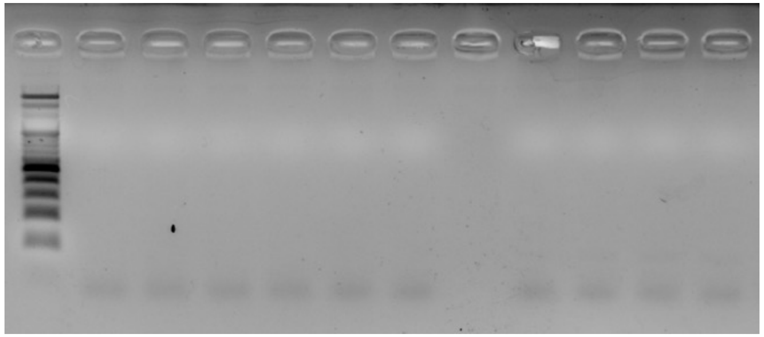

PCR GAPDH
non-ECs TIE2-TRF2DN-Tg, 25 cycles

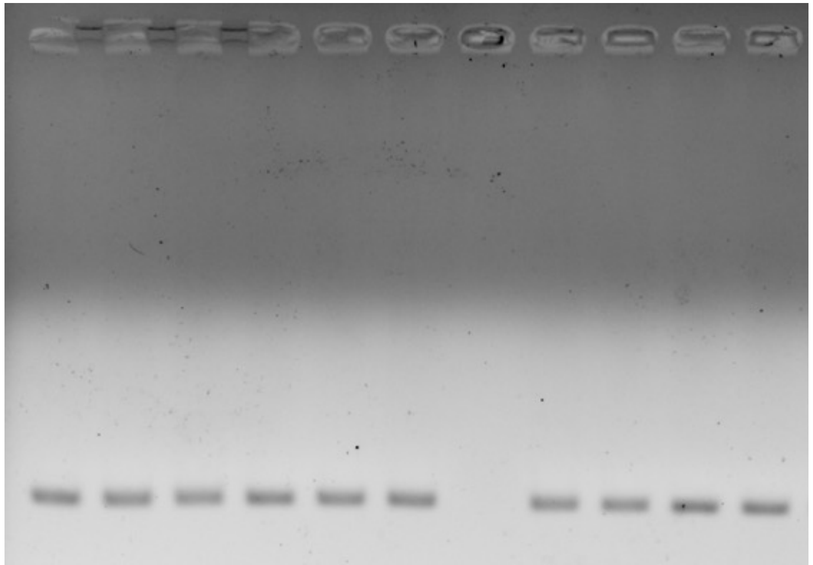

PCR GAPDH
non-ECs TIE2-TRF2DN-Tg, 25 cycles

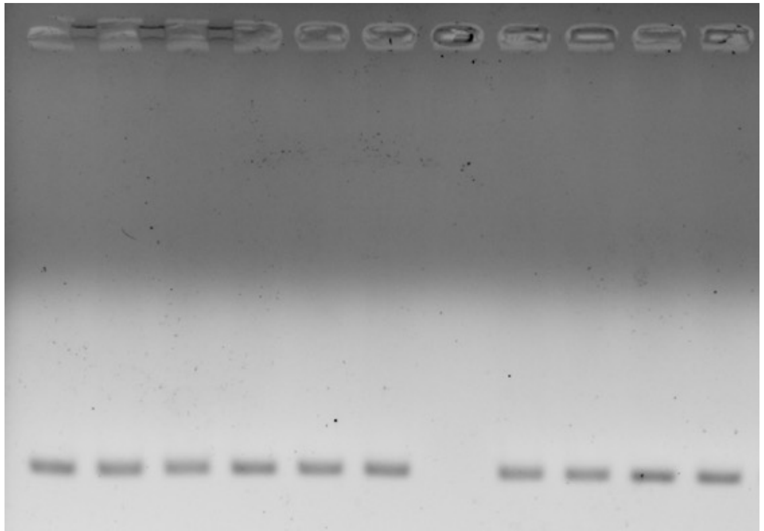

B

PCR TRANSGENE
ECs VEcad-TRF2DN-Tg, 25 cycles

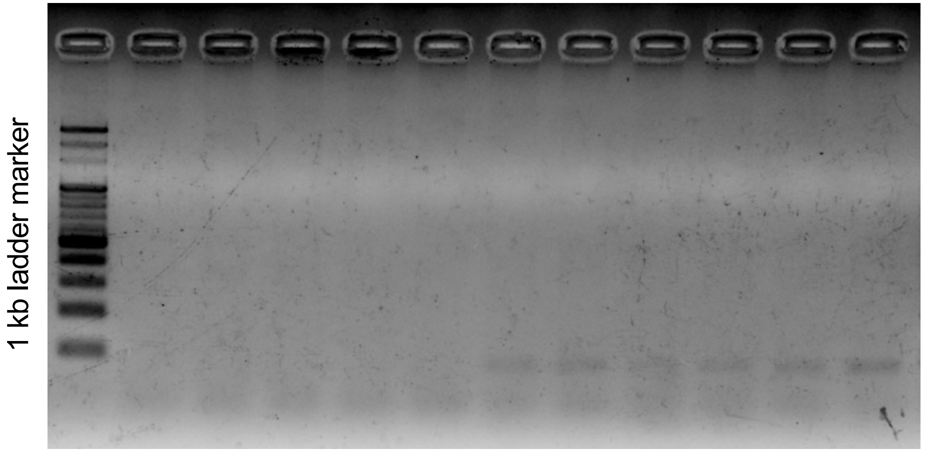

PCR TRANSGENE
non-ECs VEcad-TRF2DN-Tg, 25 cycles

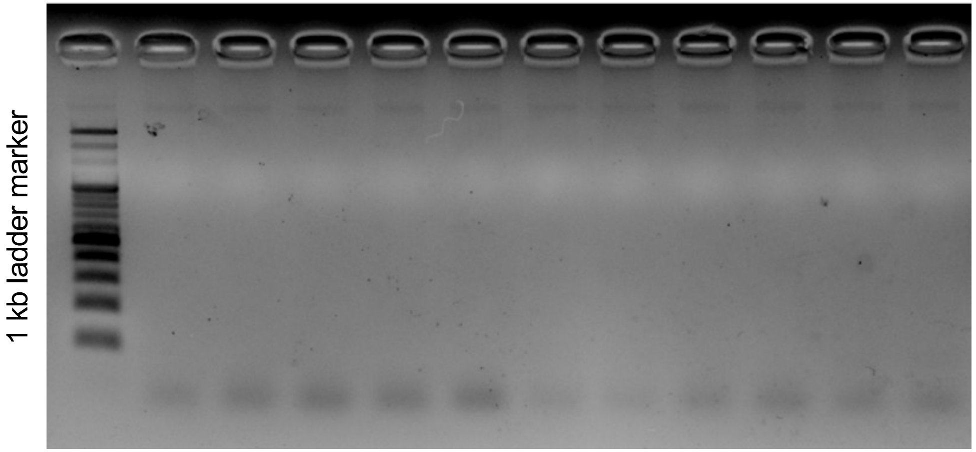

PCR GAPDH
ECs VEcad-TRF2DN-Tg, 25 cycles

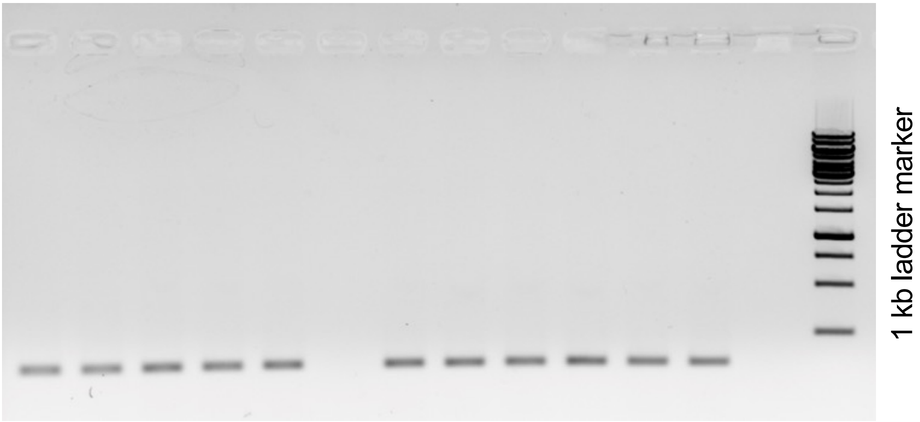

PCR GAPDH
Non – ECs VEcad-TRF2DN-Tg, 25 cycles

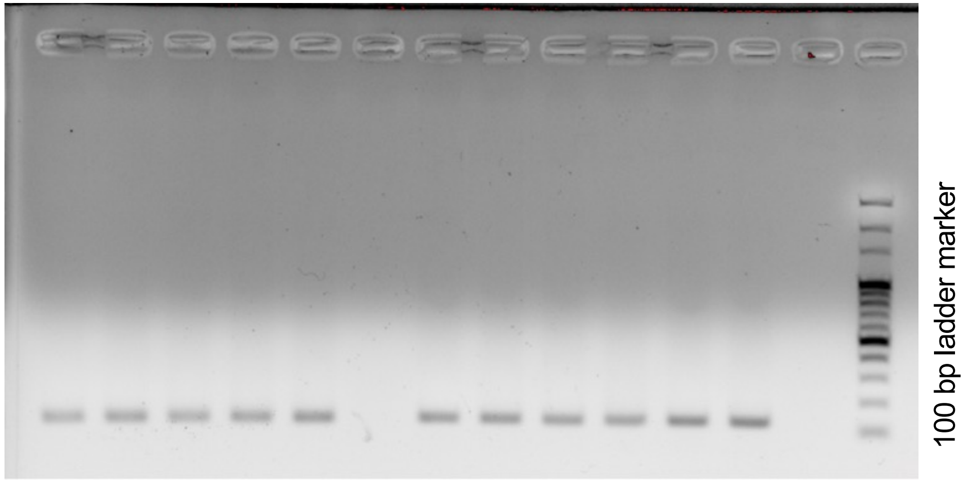

Supplementary Figure-4. Uncropped images for Supplementary Figure-1.
(**A**, **B**) Uncropped images for Supplementary Figure-1.
